# Magnetic neutron scattering from spherical nanoparticles with Néel surface anisotropy: analytical treatment

**DOI:** 10.1107/S1600576722008925

**Published:** 2022-11-04

**Authors:** Michael P. Adams, Andreas Michels, Hamid Kachkachi

**Affiliations:** a Department of Physics and Materials Science, University of Luxembourg, 162A avenue de la Faiencerie, L-1511 Luxembourg, Grand Duchy of Luxembourg; b Laboratoire PROMES CNRS UPR8521, Université de Perpignan via Domitia, Rambla de la Thermodynamique, Tecnosud, F-66100 Perpignan, France; Australian Nuclear Science and Technology Organisation, Lucas Heights, Australia

**Keywords:** magnetic neutron scattering, small-angle neutron scattering, magnetic nanoparticles, surface anisotropy, micromagnetics

## Abstract

The magnetization profile and the related magnetic small-angle neutron scattering cross section of a single spherical nanoparticle with Néel surface anisotropy are analytically investigated. A Hamiltonian is employed that comprises the isotropic exchange interaction, an external magnetic field, a uniaxial magnetocrystalline anisotropy in the core of the particle and the Néel anisotropy at the surface. Using a perturbation approach, the determination of the magnetization profile can be reduced to a Helmholtz equation with Neumann boundary condition, whose solution is represented by an infinite series in terms of spherical harmonics and spherical Bessel functions. From the resulting infinite series expansion, the Fourier transform, which is algebraically related to the magnetic small-angle neutron scattering cross section, is analytically calculated. The approximate analytical solution for the spin structure is compared with the numerical solution using the Landau–Lifshitz equation, which accounts for the full nonlinearity of the problem. The signature of the Néel surface anisotropy can be identified in the magnetic neutron scattering observables, but its effect is relatively small, even for large values of the surface anisotropy constant.

## Abstract 2

The magnetization profile and the ensuing magnetic neutron scattering signal from an inhomogeneously magnetized spherical nanoparticle with Néel surface anisotropy are derived analytically.

## 1. Introduction

Magnetic small-angle neutron scattering (SANS) is a powerful technique for investigating spin structures on the mesoscopic length scale (∼1–100 nm) and inside the volume of magnetic materials (Mühlbauer *et al.*, 2019 ▸; Michels, 2021 ▸). Recent SANS studies of magnetic nanoparticles, in particular employing spin-polarized neutrons, demonstrate that their spin textures are highly complex and exhibit a variety of nonuniform, canted or core–shell-type configurations [see *e.g.* Disch *et al.* (2012 ▸), Krycka *et al.* (2014 ▸), Hasz *et al.* (2014 ▸), Günther *et al.* (2014 ▸), Maurer *et al.* (2014 ▸), Dennis *et al.* (2015 ▸), Grutter *et al.* (2017 ▸), Oberdick *et al.* (2018 ▸), Ijiri *et al.* (2019 ▸), Bender *et al.* (2019 ▸), Bersweiler *et al.* (2019 ▸), Zákutná *et al.* (2020 ▸), Honecker *et al.* (2022 ▸) and references therein]. Surface anisotropy, vacancies or the presence of antiphase boundaries are generally considered to be at the origin of spin disorder in nanoparticles (Berger *et al.*, 2008 ▸; Wetterskog *et al.*, 2013 ▸; Nedelkoski *et al.*, 2017 ▸; Köhler *et al.*, 2021*a* ▸; Batlle *et al.*, 2022 ▸). Magnetic SANS data analysis largely relies on structural form-factor models for the cross section, borrowed from nuclear SANS, which do not properly account for the existing spin inhomogeneity inside magnetic nanoparticles or nanomagnets (NMs).

Progress in magnetic SANS theory (Honecker & Michels, 2013 ▸; Michels *et al.*, 2014 ▸; Mettus & Michels, 2015 ▸; Erokhin *et al.*, 2015 ▸; Metlov & Michels, 2015 ▸, 2016 ▸; Michels *et al.*, 2016 ▸, 2019 ▸; Mistonov *et al.*, 2019 ▸; Zaporozhets *et al.*, 2022 ▸) strongly suggests that, for the analysis of experimental magnetic SANS data, the spatial nanometre-scale variation of the orientation and magnitude of the magnetization vector field must be taken into account and macrospin-based models – assuming a uniform magnetization – are not adequate. The starting point for a proper analysis of the scattering problem is a micromagnetic continuum expression for the magnetic energy of the system. In the static case, this then leads to Brown’s equations (Brown, 1963 ▸), a set of nonlinear partial differential equations for the magnetization along with complex boundary conditions on the surface of the magnet. From these equations the Fourier image and the magnetic SANS cross section may be obtained.

In this paper, we present an analytical treatment of the magnetic SANS cross section of a spherical NM with Néel surface anisotropy (Néel, 1954 ▸). This particular form of anisotropy arises because in an NM a significant fraction of atoms belong to the surface (with no neighbours on one side), and their magnetic properties such as exchange and anisotropy can be strongly modified relative to the bulk atoms.

The manuscript is organized as follows. In Section 2, we calculate the real-space spin structure of a spherical NM using classical micromagnetic theory within the second-order perturbation approach. In Section 3, we compute the three-dimensional Fourier transform of the real-space spin structure, which directly yields the magnetic neutron scattering cross section and the pair-distance distribution function. The analytical results are benchmarked by comparing them with numerical finite difference simulations using the Landau–Lifshitz equation of motion. Finally, Section 4 summarizes the main findings of this study.

We also make reference to our accompanying numerical study (Adams *et al.*, 2022 ▸) where, in contrast to the present analytical work, the full nonlinearity of the problem is considered.

## 2. Micromagnetic theory

In the static micromagnetic approach (Brown, 1963 ▸), the magnetic configuration of a system is described by the continuous magnetization vector field **M**(**r**), which has a constant magnitude ∥**M**(**r**)∥ = *M*_0_. The saturation magnetization *M*_0_ is only a function of temperature. The normalized magnetization vector field is then defined as 

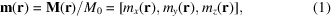

where **r** denotes the position vector. Our Hamiltonian for the NM includes the isotropic exchange interaction, the Zeeman energy, a uniaxial magnetic anisotropy for spins in the core and Néel surface anisotropy for those on the surface. In the continuum approach, it reads

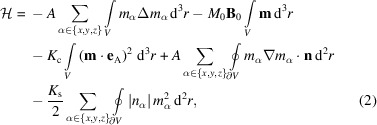

where *A* is the exchange stiffness constant, ∇ is the del operator, Δ is the Laplace operator, **B**_0_ is a constant applied magnetic field, *K*_c_ > 0 denotes the uniaxial core anisotropy constant, **e**_A_ is a unit vector specifying the arbitrary core anisotropy axis and *K*_s_ > 0 is the Néel surface anisotropy constant (Néel, 1954 ▸). 

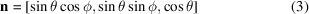

is the surface normal to the boundary of the NM, where θ and ϕ are the usual spherical angles (Garanin & Kachkachi, 2003 ▸; Kachkachi, 2007 ▸). In (2), the two surface integrals take into account the boundary conditions for the magnetization on the surface (∂*V*) of the NM of volume *V*, which result from the exchange interaction and the Néel term. The magnetodipolar energy has been ignored in the calculations because of its mathematical complexity and since it is expected to be of minor relevance for smaller-sized NMs [see the recent atomistic simulations by Köhler *et al.* (2021*b* ▸)].

For small deviations from the homogeneous magnetization state, a perturbation approach is applicable. Let **m**_0_ be the principal unit vector (average direction) associated with **m**(**r**) and let the vector function **ψ**(**r**) ⊥ **m**_0_ describe the spin misalignment. One can then write 

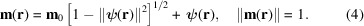

Assuming that 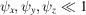 , the following second-order Maclaurin expansion in **ψ** is used to find an approximate closed-form solution for **m**(**r**): 

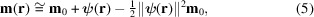

where **m**_0_ is taken as a known constant vector in subsequent calculations. We choose the orthonormal vector base (Garanin & Kachkachi, 2009 ▸), 

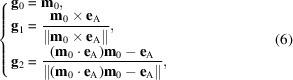

and the parametrization 

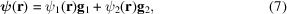

and introduce the dimensionless coordinates **ξ** = **r**/*R* (with ξ = ∥**ξ**∥ = *r*/*R*), where **r** is the position vector, 

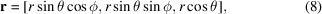

and *R* denotes the radius of the NM. The minimization of the Hamiltonian (2) then leads to the well known Helmholtz equation with Neumann boundary conditions on the unit sphere (Kachkachi, 2007 ▸; Garanin & Kachkachi, 2003 ▸), 

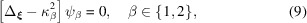




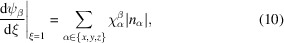

where the constants are defined as

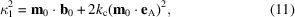




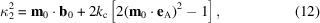




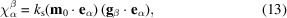

with the dimensionless quantities 

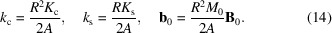

The **e**_α_ (with α = *x*, *y*, *z*) in (13) denote the unit vectors of the Cartesian laboratory coordinate frame (in which **n** and **r** are defined). We emphasize that there are only two independent differential equations for **ψ**, which is a consequence of the constraint ∥**m**(**r**)∥ = 1.

In our graphical representations, we will frequently use the following values: *k*_c_ = 0.1 and *k*_s_ = 3.0, which (using *R* = 5 nm and *A* = 10^−11^ J m^−1^) correspond to *K*_c_ = 80 kJ m^−3^ and *K*_s_ = 12 mJ m^−2^ (Gradmann, 1986 ▸; O’Handley, 2000 ▸; Batlle *et al.*, 2022 ▸). For *M*_0_ = 1.7 × 10^6^ A m^−1^, the relation between *b*_0_ (dimensionless) and the external field is *B*_0_ = (8/17)*b*_0_ × 1 T.

The fundamental solution of the homogeneous Helmholtz equation (9) is well known (Weber & Arfken, 2003 ▸; Riley *et al.*, 2006 ▸). Its non-singular part can be expressed in spherical coordinates as an infinite series in terms of spherical harmonics *Y*_ℓ*m*_(θ, ϕ) and spherical Bessel functions of the first kind *j*_*n*_(iκ_β_ξ), 

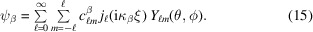

The imaginary number ‘i’ in the argument of the spherical Bessel function is due to the negative sign in the Helmholtz equation (9). The expansion coefficients 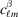 are obtained from the Neumann boundary condition (10) using the method of least squares (see Appendix *A* [App appa]). From Appendix *A* [App appa] it is seen that the zero-order term with ℓ = 0 vanishes. This physically makes sense, since the spin misalignment in our model is caused by the Néel surface anisotropy and thus, for symmetry reasons, there is no misalignment at the centre of the NM, *i.e.* ψ_β_(ξ = 0, θ, ϕ) ≡ 0. By contrast, the largest spin misalignment is found at the boundary of the NM, *i.e.* ξ = 1. Further, we find that the coefficients 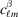 vanish in the case of odd ℓ and *m*, while they are real valued and even with respect to the index *m*, *i.e.* 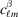 = 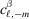 . Taking these properties into account, one can conveniently express the solution in terms of the associated Legendre polynomials 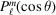 with ℓ = 2ν and *m* = 2μ {note that we use the convention that 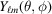 = 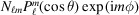 [p. 378 (14.30.1) of Olver *et al.* (2010 ▸)]},

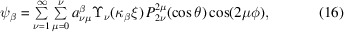

where we define [compare pp. 624–626 of Weber & Arfken (2003 ▸)] 

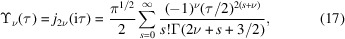

and the expansion coefficients are given by 

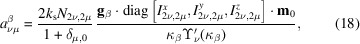

with 

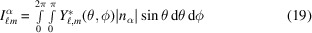

and 

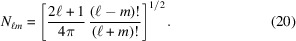

In (18), δ_μ, 0_ is the Kronecker delta function, diag[…] denotes a 3 × 3 diagonal matrix and 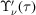 is the first-order derivative of (17) with respect to τ. For some small values of ℓ and *m*, the exact solutions of the integrals 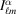 are listed in Table 1 in Appendix *B* [App appb].

From (18) it is seen that the functions ψ_β_ depend linearly on *k*_s_, such that for *k*_s_ = 0 the magnetization of the NM is homogeneous (as expected). Since we assume that 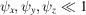 , it is clear that the validity of our solution is restricted to a finite range 0 ≤ *k*_s_ ≤ *k*_s, max_. Taking only the terms with ν = 1 into account (corresponding to ℓ = 2), the remaining (second-order) expression reads 

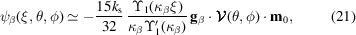

where 

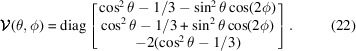

A reasonable approximation for small κ_β_ in (21) is obtained by taking into account the first two terms in the infinite series (17) for ϒ_ν_(τ). This results in the following expression [compare (21)]: 

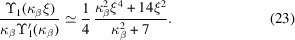

In the limit κ_β_→0 (small **B**_0_ and small *K*_c_), this expression reduces to a quadratic function in ξ, 

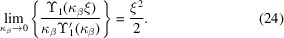

The case of an infinite applied magnetic field **B**_0_, or of a strong uniaxial core anisotropy [compare (11) and (12)], corresponds to the limit 

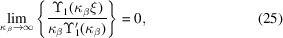

which recovers the expected result of zero spin misalignment. Note that the limit κ_β_→∞ is only obtained using all terms of the infinite series (17).

Of particular interest is the behaviour of ψ_β_ as a function of the radius *R* of the NM. Inspecting the Hamiltonian (2), it becomes clear that the surface anisotropy energy scales as *R* ^2^, while the uniaxial core anisotropy energy scales as *R* ^3^. Since the core and surface anisotropies act in opposite ways (trying to make the spin structure more homogeneous and more inhomogeneous, respectively), we see that an increasing radius *R* corresponds to a decreasing ψ_β_. This behaviour reflects the NM’s surface-area-to-volume ratio. With (21) it is not possible to make any prediction in this regard, because until this point we have not included the principal unit vector **m**_0_ in the minimization of the Hamiltonian. Generally, **m**_0_ is a function of *k*_s_, *k*_c_, **b**_0_ and **e**_A_.

**Figure 1:**
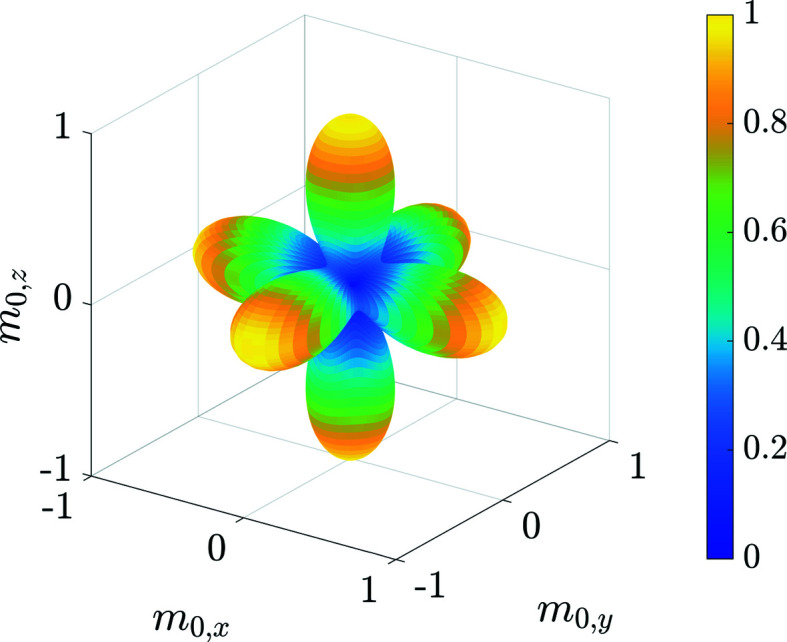
The normalized effective energy potential of the Néel surface anisotropy as a function of the Cartesian components of the average magnetization vector **m**_0_ = [sinβcosα, sinβsinα, cosβ], computed via numerical integration of the surface contribution in (2) and using the second-order approximation (21). Parameters are **e**_A_ = [0, 0, 1], **b**_0_ = **0**, *k*_c_ = 0.1 and *k*_s_ = 3.0. The minima of the Néel surface contribution are in this case along the cubic space diagonals **m**_0_ = [±1, ± 1, ± 1]/(3^1/2^), while the maxima correspond to the Cartesian axes ± **e**_*x*_, ± **e**_*y*_, ± **e**_*z*_. The effective energy potential has cubic symmetry and is approximately proportional to a function of the type 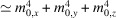 [see also Garanin & Kachkachi (2003 ▸)].

In the special case when the uniaxial anisotropy axis and the applied magnetic field are both directed parallel to the *z* axis (**e**_A_ = [0, 0, 1] and **b**_0_ = [0, 0, *b*_0_]), the principal unit magnetization vector may be written as

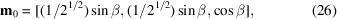

where 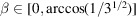 . This choice is justified by the effective cubic symmetry of the Néel anisotropy as shown in Fig. 1 ▸. This result was already predicted by Garanin & Kachkachi (2003 ▸). The solutions for ψ_1, 2_(ξ, θ, ϕ) [using the particular **m**_0_ (26)] then read 

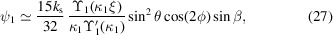




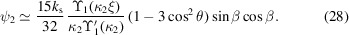

**Figure 2:**
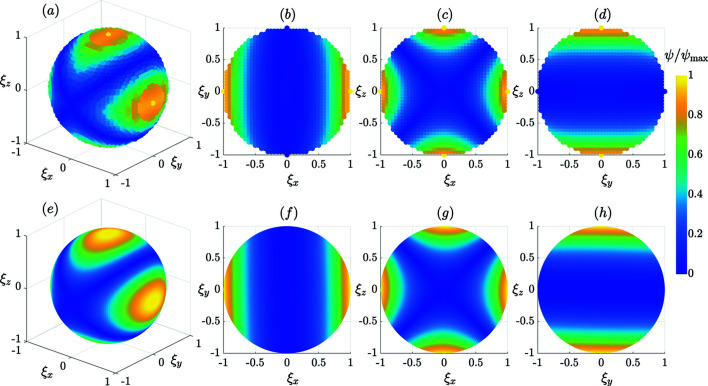
A comparison between the numerical solution using the Landau–Lifshitz equation (upper row) and the second-order analytical solution (21) for 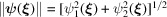 (lower row). (*a*) and (*e*) show ∥**ψ**∥ on the boundary surface (ξ = 1), while (*b*)–(*d*) and (*f*)–(*h*) display selected planar cuts in (ξ_*x*_, ξ_*y*_, ξ_*z*_) space. The following parameters are used: **e**_A_ = **e**_*z*_, **b**_0_ = [0.4, 0, 0.4] (*B*_0_ ≅ 266 mT), *k*_c_ = 0.1, *k*_s_ = 3.0 and **m**_0_ = [sinβcosα, sinβsinα, cosβ], where α = 0° and β = 40°.

**Figure 3:**
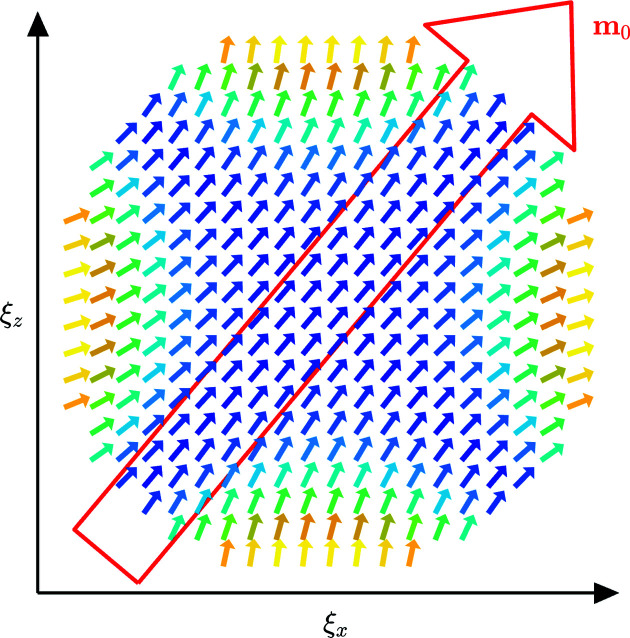
The real-space spin structure in the ξ_*x*_ξ_*z*_ plane computed using (4) and (21). Parameters are the same as in Fig. 2 ▸. The external field *B*_0_ ≃ 266 mT is applied in the ξ_*x*_ξ_*z*_ plane and inclined by an angle of β = 40° relative to the ξ_*z*_ axis [compare with Garanin & Kachkachi (2003 ▸)].

In Fig. 2 ▸, the analytical solution (21) (lower row) is compared with the numerical solution based on the Landau–Lifshitz equation 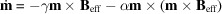 (upper row) (Bertotti, 1998 ▸), where γ is the gyromagnetic ratio, α is the damping constant and the dot denotes the first-order time derivative [see our numerical study in the accompanying paper (Adams *et al.*, 2022 ▸) for further details]. Shown is the vector norm of the **ψ**(**ξ**) function scaled to its maximum value. From Fig. 2 ▸ it is seen that our analytical approximation is in qualitative agreement with the results from the numerical simulation. The corresponding real-space spin structure **m**(**ξ**) is displayed in Fig. 3 ▸, where the surface spin disorder becomes clearly visible.

It is also instructive to compare our solution (21) with that obtained using the Green’s function approach (Garanin & Kachkachi, 2003 ▸; Kachkachi, 2007 ▸). In particular, for ξ located close to the surface, where the maximum spin misalignment with respect to **m**_0_ occurs, the Green’s function method yields the following approximate expression: 

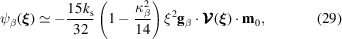




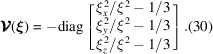

This expression is also found when (21) is expanded in κ_β_ at the surface of the NM (ξ = 1).

While the infinite series approach using spherical harmonics and spherical Bessel functions yields an exact solution of the Helmholtz equation, the Green’s function approach provides an approximate explicit expression of ψ_β_ in terms of the coefficients κ_β_. Indeed, as was shown by Kachkachi (2007 ▸), in the presence of core anisotropy Green’s function as the kernel of the Helmholtz equation is only obtained as a perturbative series in κ_β_. As such, (29) is restricted to small values of κ_β_, *i.e.* assuming that the core anisotropy and applied magnetic field are much smaller than the exchange coupling. This is manifest in (29) by the presence of the factor 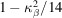 which implies that the contribution of spin misalignment may diverge when κ_β_ is too large (*i.e.* for a strong field and/or large core anisotropy).

## 3. Magnetic SANS cross section

**Figure 4:**
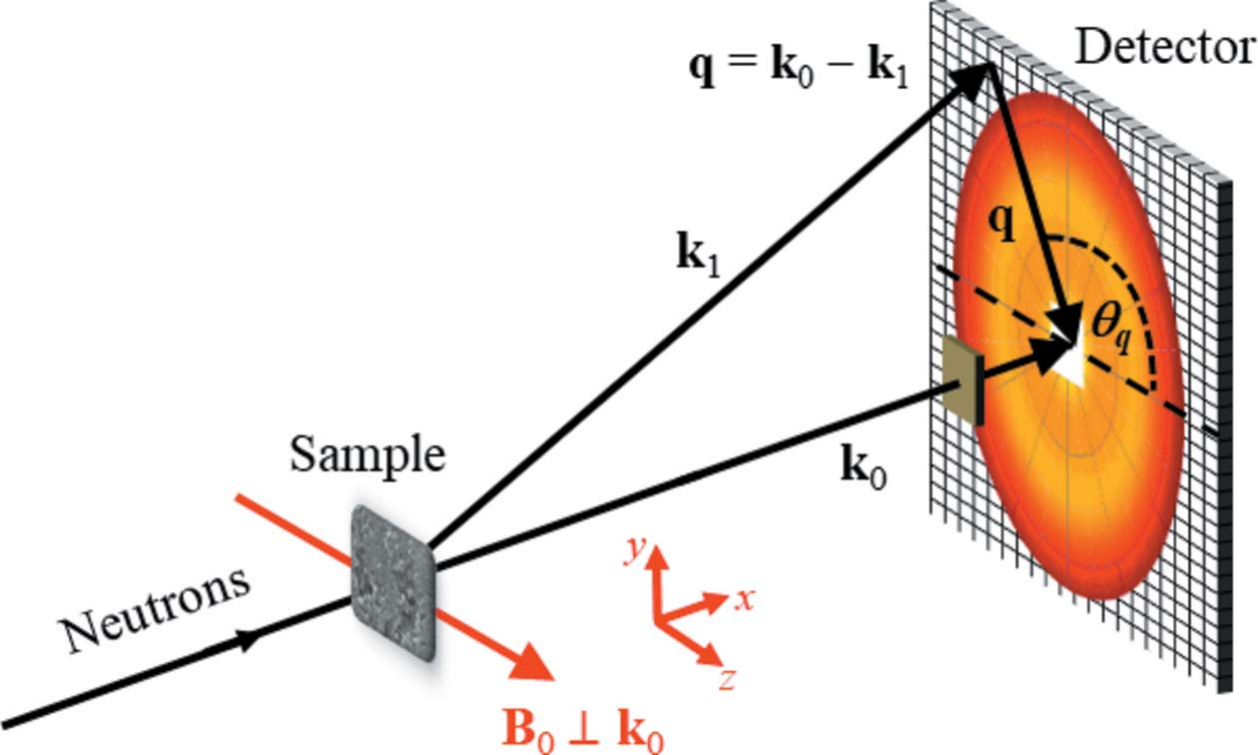
A sketch of the perpendicular scattering geometry (**B**_0_ ⊥ **k**_0_). The scattering vector **q** corresponds to the difference between the wavevectors of the incident (**k**_0_) and scattered (**k**_1_) neutrons. The angle θ_*q*_ specifies the orientation of **q** on the detector. In the small-angle approximation, the component of **q** along **k**_0_ is neglected.

The quantity of interest in experimental SANS studies is the elastic magnetic differential scattering cross section dΣ_M_/dΩ, which is usually recorded on a two-dimensional position-sensitive detector. For the most commonly used scattering geometry in magnetic SANS experiments, where the applied magnetic field **B**_0_ ∥ **e**_*z*_ is perpendicular to the wavevector **k**_0_ ∥ **e**_*x*_ of the incident neutrons (see Fig. 4 ▸), dΣ_M_/dΩ (for un­polarized neutrons) can be written as (Mühlbauer *et al.*, 2019 ▸) 

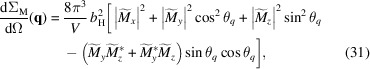

where *V* is the scattering volume and *b*_H_ = 2.91 × 10^8^ A^−1^ m^−1^ is the magnetic scattering length in the small-angle regime (the atomic magnetic form factor is approximated by 1, since we are dealing with forward scattering). 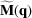 = 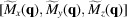 represents the magnetization vector field **M**(**r**) in Fourier space, θ_*q*_ denotes the angle between the scattering vector **q** and **B**_0_ (not to be confused with the polar angle θ defined above), and the asterisk * stands for the complex conjugate. Note that in the perpendicular scattering geometry the Fourier components are evaluated in the plane *q*_*x*_ = 0.

The Fourier transform of the three-dimensional magnetization vector field (with a tilde above the symbol) is defined as 

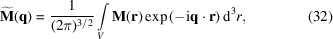




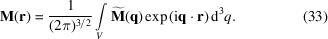

For subsequent calculations, we introduce the following dimensionless quantities: 

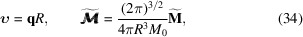

and we express the dimensionless scattering vector in spherical coordinates as 

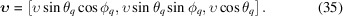

Next, in (32) we use the following first-order approximation for the real-space magnetization vector **m**(**ξ**) [see (5) and (7)]: 

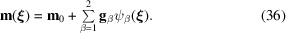

As shown in Appendix *C* [App appc], the final expression for the Fourier transform of the magnetization is then given by 

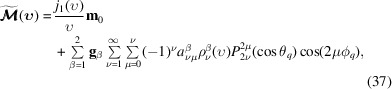

where 

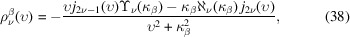




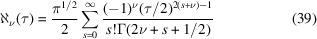

and ϒ_ν_(κ_β_) is given by (17). The zero-order term ∝ *j*_1_(υ)/υ in (37) represents the form factor of a homogeneously magnetized sphere (Michels, 2021 ▸). In the limiting case of an infinite applied magnetic field, which is equivalent to the limit κ_β_→∞, the additional terms [second line in (37)] vanish [compare with (25)] and the spherical form factor remains. On the other hand, if *k*_s_ = 0, the additional terms also vanish because, from the physical point of view, the Néel surface anisotropy cancels and from (18) we know that the coefficients 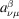 are linear in *k*_s_. Taking only the terms with ν = 1 into account and setting ϕ_*q*_ = π/2 (υ_*x*_ = 0), corresponding to the scattering geometry where the applied magnetic field **B**_0_ ∥ **e**_*z*_ is perpendicular to the wavevector **k**_0_ ∥ **e**_*x*_ of the incident neutrons (Fig. 4 ▸), the expression for 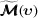 can be written as [compare with (21)]

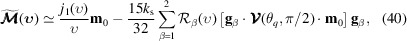

where the radial function is 

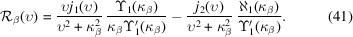




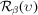
 can be approximated for small κ_β_ and, when only terms up to *s* = 1 in the infinite series (17) and (39) are kept,

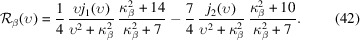

For small υ values, one finds the limit 

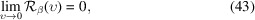

which is consistent with 

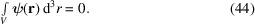

This can be seen by inspecting the definition of the Fourier transform in (32). Note that for *q*→0 the Fourier transform is proportional to the average of the magnetization vector field **M** and the maximum of this average is given by the homogeneous magnetization state. Using this result, the υ→0 limit for the first-order approximation in **ψ** of the Fourier transform of the magnetization yields

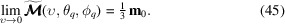

Beyond the linear approximation in **ψ**, a non-vanishing term appears in 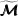 in the limit υ→0, which reduces the Fourier components relative to the homogeneous magnetization state. In the second order in **ψ**, the result is [compare (5)]

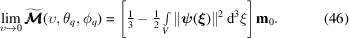

Using (34) and 

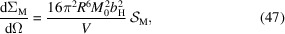

the dimensionless two-dimensional magnetic SANS cross section 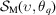 can be straightforwardly obtained as [compare (31)] 

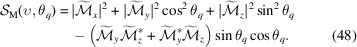

In the limit *k*_s_→0, the resulting cross section from (37) is 

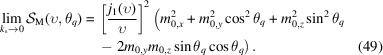

Relation (49) nicely demonstrates that, depending on the orientation of the uniformly magnetized particle, different angular anisotropies become visible on the detector. For **m**_0_ ∥ **e**_*x*_ (*i.e.* *m*_0*y*_ = *m*_0*z*_ = 0) the scattering pattern is isotropic, while it exhibits a 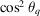 ( 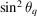 ) type shape when **m**_0_ ∥ **e**_*y*_ (**m**_0_ ∥ **e**_*z*_).

**Figure 5:**
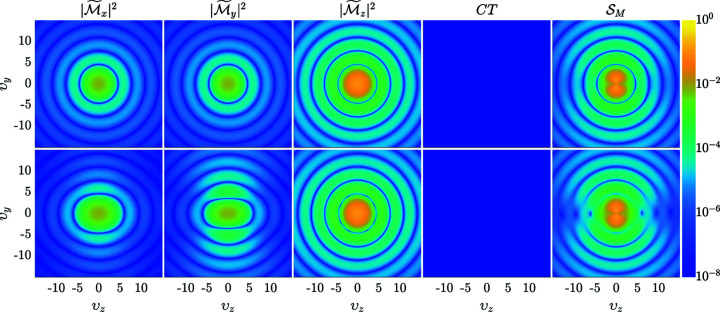
Results for the two-dimensional Fourier components 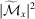 , 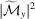 , 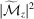 , CT = 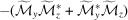 and for the total magnetic SANS cross section 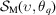 [(48)] using expression (40). The upper row shows the results taking into account only the zero-order term in (40), which corresponds to the case of a homogeneously magnetized particle. The lower row displays the results when the second-order term (ν = 1) in (40) is taken into account. The parameters are **e**_A_ = **e**_*z*_, **b**_0_ = 0.1**e**_*z*_ (*B*_0_ ≃ 48 mT), *k*_c_ = 0.1, *k*_s_ = 3 and **m**_0_ = [sinβcosα, sinβsinα, cosβ]. Note that υ_*y*_ and υ_*z*_ denote the dimensionless components of the scattering vector [compare equation (34)]. Since the Néel surface anisotropy effectively has a cubic symmetry (see Fig. 1 ▸), we average 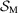 over the angles α = (45°, 135°, 225°, 315°) and β = 20°. A logarithmic colour scale is used.

Fig. 5 ▸ shows 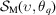 along with the contribution of the individual Fourier components to (48). The upper row in Fig. 5 ▸ presents the results taking into account only the zero-order term [*j*_1_(υ)/υ]**m**_0_ from (40), while in the lower row the second-order term (ν = 1) is additionally included. Since the zero-order term represents the case of a homogeneously magnetized NM, this comparison provides useful insights about the impact of the Néel surface anisotropy on the magnetic SANS cross section. In the case of a uniformly magnetized NM (upper row) the Fourier components 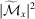 , 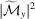 and 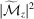 are isotropic (rotational symmetry), while including the second-order terms (lower row) leads to anisotropic behaviour of the transverse components 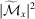 and 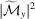 . The cross term (CT) averages to zero for both situations and the dominant contribution to the magnetic SANS cross section (for the parameters chosen in Fig. 5 ▸) is given by the 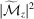 component. Therefore, it may be concluded that the impact of the Néel surface anisotropy on 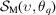 is relatively small. By comparing the 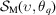 from the upper and lower rows, it is seen that by including the Néel surface anisotropy the circular symmetry of the zeros of 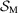 (deep-blue colours) is broken. This feature becomes more clearly visible by analyzing the azimuthal average of 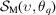 , which is readily computed as

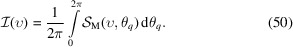

In the limit *k*_s_→0, the azimuthal average corresponding to (49) is

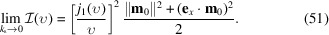

We have also calculated the pair-distance distribution function 

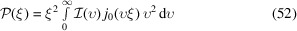

and the correlation function 

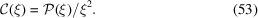

In the limit *k*_s_→0, the pair-distance distribution and the correlation function corresponding to (51) are

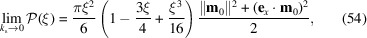




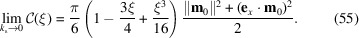

**Figure 6:**
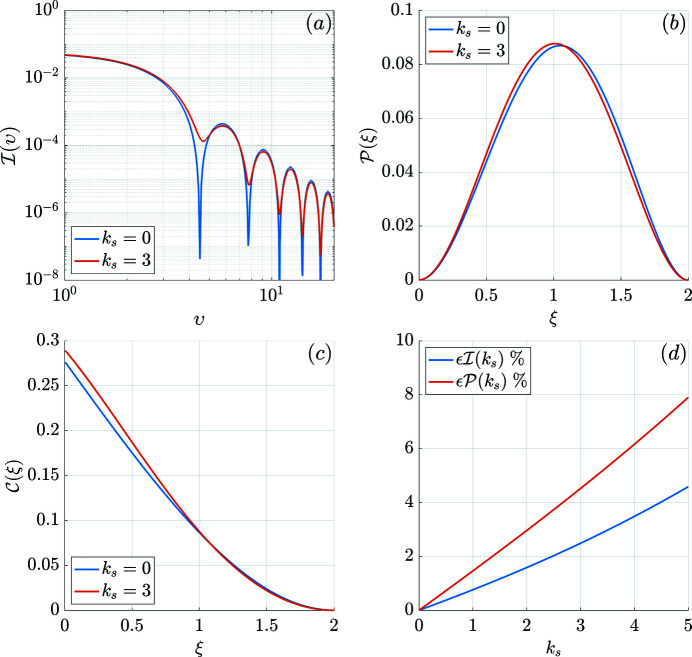
Results for (*a*) the azimuthally averaged SANS cross section 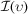 , (*b*) the pair-distance distribution function 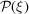 , (*c*) the correlation function 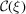 , and (*d*) the quantities 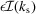 [(56)] and 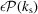 [(57)] (*B*_0_ ≅ 48 mT). For the homogeneous case [blue curves in panels (*a*)–(*c*)] the surface anisotropy is set to *k*_s_ = 0, and for the inhomogeneous case [red curves in panels (*a*)–(*c*)] we use the same parameters as in Fig. 5 ▸. The functional dependence of 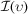 , 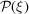 and 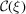 for the uniformly magnetized particle all correspond to the analytically well known cases, *i.e.* (51), (54) and (55).

These functions are displayed graphically in Fig. 6 ▸. Due to the surface-anisotropy-induced spin disorder, the form-factor extrema of 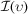 [Fig. 6 ▸(*a*)] are damped and shifted slightly to larger *q* values [*i.e.* smaller structures; compare the first minimum of 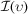 for *k*_s_ = 3]. Moreover, as already observed in numerical micromagnetic continuum simulations (Vivas *et al.*, 2017 ▸, 2020 ▸), the oscillations are damped for the case of surface spin disorder, which mimics the effect of a particle-size distribution or of instrumental resolution. In agreement with this observation is the finding that the maximum of the 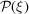 function [Fig. 6 ▸(*b*)] appears at smaller distances ξ than in the homogeneous case. Likewise, due to spin disorder, the 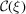 function [Fig. 6 ▸(*c*)] exhibits a larger amplitude (Mettus & Michels, 2015 ▸).

To analyze the role of the surface anisotropy more quantitatively, we have computed the following quantities, which describe the deviation of the one-dimensional SANS cross section and of the pair-distance distribution function from the homogeneous particle case: 

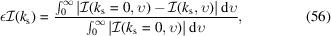




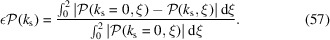

Fig. 6 ▸(*d*) depicts both 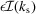 and 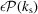 as a function of *k*_s_. The difference is only of the order of a few percent, which suggests that the effect of surface anisotropy on the SANS observables is relatively weak within the present analytical approximation: see our accompanying numerical work (Adams *et al.*, 2022 ▸), which takes into account the full non­linearity of the micromagnetic equations. However, this is only true for the magnetic interactions considered here. Taking into account the anisotropic and long-range dipole–dipole interaction and the asymmetric Dzyaloshinskii–Moriya interaction will very likely result in more inhomogeneous spin structures and in larger deviations from the macrospin model (Vivas *et al.*, 2017 ▸, 2020 ▸; Pathak & Hertel, 2021 ▸). Likewise, for NMs of elongated shapes, the surface anisotropy renders an additional first-order contribution to the effective energy (Garanin & Kachkachi, 2003 ▸), in addition to the second-order cubic contribution discussed above. This new shape-induced contribution could also lead to an enhancement of the spin misalignment. The analytical calculations presented here provide a general framework for future studies of more complicated (anisotropic) magnetic interactions. The approach can be straightforwardly adapted to other particle shapes such as a circular planar disc.

## 4. Conclusions

We have analytically computed the magnetization distribution and the ensuing magnetic SANS cross section of a spherical nanoparticle. Our micromagnetic Hamiltonian takes into account the isotropic exchange interaction, an external magnetic field, a uniaxial anisotropy for the particle’s core and Néel anisotropy on its boundary. The resulting Helmholtz equation has been solved by expanding the real-space magnetization in terms of spherical Bessel functions and spherical harmonics. The central results are the infinite series (16) and its second-order expansion (21) for the real-space magnetization, and the corresponding Fourier transforms (37) and (40). Using these expressions, the two-dimensional magnetic SANS cross section 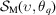 , the azimuthally averaged SANS signal 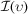 , and the correlation functions 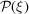 and 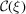 have been obtained and compared with the case of a homogeneous spin configuration (uniform magnetization vector field). The signature of Néel surface anisotropy (of constant *k*_s_) has been identified in all of these functions. However, its effect is relatively small, even for large values of *k*_s_. Taking into account the magnetodipolar and/or the Dzyaloshinskii–Moriya interaction, or shape asymmetry, will probably result in configurations with stronger spin misalignment (*e.g.* in vortex-type textures or skyrmions) and thereby in more prominent signatures in the SANS cross section and correlation function. These interactions are beyond the scope of the current analytical approach and will be considered in our future (numerical) work.

## Supplementary Material

Mathematica script for the calculation of the integrals, using direct symbolic integration. DOI: 10.1107/S1600576722008925/in5070sup1.txt

Mathematica script for the calculation of the integrals, using the analytical simplifications described in the appendix. DOI: 10.1107/S1600576722008925/in5070sup2.txt

## Figures and Tables

**Table:** 

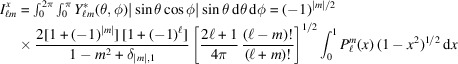							
	*m*						
ℓ	0	± 1	± 2	± 3	± 4	± 5	± 6
0	π^1/2^						
1	0	0					
2	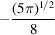	0	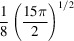				
3	0	0	0	0			
4	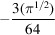	0	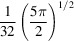	0	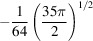		
5	0	0	0	0	0	0	
6	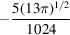	0	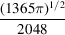	0	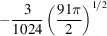	0	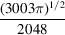

**Table:** 

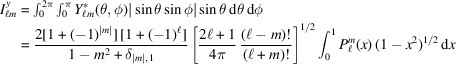							
	*m*						
ℓ	0	± 1	± 2	± 3	± 4	± 5	± 6
0	π^1/2^						
1	0	0					
2	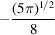	0	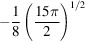				
3	0	0	0	0			
4	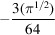	0	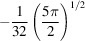	0	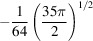		
5	0	0	0	0	0	0	
6	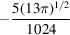	0	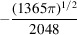	0	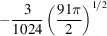	0	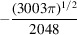

**Table:** 

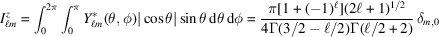							
	*m*						
ℓ	0	± 1	± 2	± 3	± 4	± 5	± 6
0	π^1/2^						
1	0	0					
2	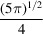	0	0				
3	0	0	0	0			
4	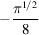	0	0	0	0		
5	0	0	0	0	0	0	
6	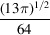	0	0	0	0	0	0

## Appendix A Solution of the boundary value problem of the Helmholtz equation

The coefficients  in the fundamental solution (15) of the Helmholtz equation (9) must be determined such that the Neumann boundary condition (10) is satisfied. For this purpose, we use the method of least squares, where we make use of the orthogonality properties of the spherical harmonics *Y*_ℓ*m*_(θ, ϕ). The normal derivative of (15) at the surface of the NM (ξ = 1) is

where

Our goal is now to minimize the following error functional with respect to the coefficients  :

The minimum of this error functional is found from the condition that the partial derivatives of  with respect to  vanish,

where * denotes the complex conjugate. Using the orthogonality relation (62) [p. 378 (14.30.8) of Olver *et al.* (2010 ▸)] and by defining the integral  by (63),

the solution of (61) can be written as

Alternatively, again using the indices ℓ and *m*, expressing the coefficient  as in (13) and using the matrix vector product, the coefficients  can be more explicitly written as

For some low orders of ℓ and *m*, the exact solutions of the integrals  are presented in Appendix *B* [App appb].

From (65) several conclusions can be drawn. First, the zero-order term (ℓ = *m* = 0) in (15) vanishes, which can easily be shown by rewriting the coefficient  as

and this is due to the orthogonality of **m**_0_ and **g**_β_ with β ∈ {1, 2} [see (6)]. Moreover, we see that

which is a consequence of the behaviour of the spherical Bessel functions of the first kind at the origin. Since the *j*_ℓ_ with ℓ ≥ 1 all vanish at the origin ξ = 0, this implies that the total ψ_β_ also vanishes at the origin. Note that *j*_0_(0) = 1 but does not contribute to ψ_β_ since  = 0. From the physical point of view this makes sense, since the spin misalignment is caused by the Néel surface anisotropy and thus, for symmetry reasons, there is no spin disorder at the centre of the spherical NM; the highest misalignment is found at its surface. Secondly, in Table 1 in Appendix *B* [App appb] it is seen that the coefficients  vanish for odd ℓ or *m* and that  =  , so that the expansion coefficients also exhibit this symmetry,

Taking these properties into account, one can express the solution (15) more conveniently in terms of the associated Legendre polynomials  with ℓ = 2ν and *m* = 2μ {note that we use the convention that  =  ×  [p. 378 (14.30.1) of Olver *et al.* (2010 ▸)]},

where ϒ_ν_(τ) is defined in (17) and  in (18).

The infinite series in (18) is a consequence of the relation between the spherical Bessel functions of the first kind and the ordinary Bessel functions of the first kind [p. 262 (10.47.3) of Olver *et al.* (2010 ▸)],

and the well known series representation [p. 262 (10.47.3) of Olver *et al.* (2010 ▸)]

## Appendix B Integral coefficients

The integrals  can be simplified, since both *n*_α_ and  are separable functions in θ and ϕ. By rewriting the spherical harmonics in terms of complex exponentials and associated Legendre polynomials, and by using the definition of the Cartesian components of the surface normal vector **n** from (3) {note that we use the convention that  =  [p. 378 (14.30.1) of Olver *et al.* (2010 ▸)]}, the integrals are expressed as follows:

where

The integrals  ,  and  are solvable straightforwardly using Euler’s formula for the complex exponential and by splitting the region of integration according to the absolute values of the trigonometric functions. In the denominator of  and  we have included the Kronecker delta δ_|*m*|,1_ to take account of the cases *m* = ±1. It is common to express the integrals  and  by the substitution *x* = cosθ (d*x* = −sinθ dθ),

Using  , we need only compute  such that the associated Legendre polynomials in  are reduced to the Legendre polynomials with one index only [p. 352 of Olver *et al.* (2010 ▸)]. By considering the symmetry properties of the Legendre polynomials it becomes clear that the integral must vanish for odd ℓ and can be simplified for even ℓ in the following way:

The closed form of  is then found in terms of the Gamma function [p. 771 (7.126.1) of Gradshteyn & Ryzhik (2007 ▸)],

The overall solution for  is then written as

Since we only have to calculate  for even *m* [because  and  include the term 1 + (−1)^|*m*|^], we may use the index *m* = 2μ and, by exploiting the parity of the associated Legendre polynomials  =  , we find

From these results it is seen that the integrals  with α ∈ {*x*, *y*, *z*} vanish for odd ℓ and *m* [note that in (86) this is only the case for *m* = 2μ]. For the remaining integrals  in (86), where ℓ = 2ν, we do not give an expression in closed form. However, one must exist in terms of the Gamma function or the Beta function, since [p. 324 (3.251.2) of Gradshteyn & Ryzhik (2007 ▸)]

where B(·, ·) is the Beta function (Euler integral), and the associated Legendre functions  of even order and degree are true polynomials, as seen for example from the related Rodrigues formula [p. 360 (14.7.14) of Olver *et al.* (2010 ▸)]. We used *Mathematica* (Wolfram Inc.) to determine the integrals up to the sixth order in ℓ (see Table 1 ▸).

## Appendix C Derivation of the Fourier transform of the magnetization

The Fourier transform of the magnetization vector field **M**(**r**) is written as

In the following, we will use dimensionless quantities. For this purpose, we define the dimensionless scattering vector **υ** = **q** *R*, where *R* is the radius of the nanomagnet, the dimensionless position vector **ξ** = **r**/*R* and the dimensionless magnetization vector **m** = **M**/*M*_0_, where *M*_0_ is the saturation magnetization. Substituting in (88) results in

The dimensionless Fourier transform  is then defined as

with

The next step consists of calculating the Fourier integral of the first-order approximation (36) of the magnetization vector **m**. Since (36) is formulated in dimensionless spherical coordinates ξ, θ, ϕ, it is convenient to express the scattering vector **υ** in spherical coordinates as well,

so that the plane-wave expansion of the complex exponential can be used (Jackson, 1999 ▸),

The Fourier integral (90) is then expanded into the following infinite series:

where

We now use the infinite series (15) for ψ_β_ to express the first-order approximation of the magnetization, which leads to

Since the integral transform (96) is linear, each term in (97) can be separately transformed. For the zero-order term, we obtain

This result is well known to the neutron-scattering community as the spherical form factor, corresponding to a uniformly magnetized spherical particle (Michels, 2021 ▸). In the second step, we carry out the integration of the higher-order terms from (97). The radial and angular parts in the higher-order terms of (97) are multiplicative, such that the volume integral (96) is separable into

As an intermediate result, the integral (96) is then rewritten as

The integral (100) directly corresponds to the orthogonality relation of the spherical harmonics and thereby we have  =  [p. 378 (14.30.8) of Olver *et al.* (2010 ▸)]. Due to the term δ_ℓ*k*_ in  and since  and  are multiplicative in (101), the following spherical Hankel transform results:

In order to calculate these integrals, we replace [using (70)] the spherical Bessel functions of the first kind *j*_*k*_(·) with the ordinary Bessel functions of the first kind *J*_ι_(·). This yields

where

is the Hommel integral. The result for the integral  can be found in the textbook by Gradshteyn & Ryzhik (2007 ▸) [p. 664 (6.521.1)] and is written as

Again, upon applying relation (70), we write the solution for  more conveniently in terms of spherical Bessel functions of the first kind as

Now that the integrals have been obtained, we have to substitute (101) in (95). The term on the first line of (101) only accounts for *k* = 0 and *n* = 0 in (95). Therefore, the contribution of this term to  is [*j*_1_(υ)/υ]**m**_0_, because *Y*_00_(θ, ϕ) = 1/(4π)^1/2^. Since  , the final result is

Using the properties of the coefficients  studied in Appendix *A* [App appa], we reformulate (107) in terms of the associated Legendre polynomials, the indices *k* = 2ν and *n* = 2μ, and the coefficients  :

where the radial function is given by

Using once again expression (70) and the well known series (71) for the Bessel functions of the first kind, we redefine the spherical Bessel functions of imaginary arguments [as they appear in (109)] as

such that it becomes clear that  is a purely real-valued function. The radial function is then rewritten as

By comparing this result with (102), we find the following pair of spherical Hankel transforms,

By comparing the result of (108) with (16) it becomes clear that the angular part is (due to the orthogonality relation of the spherical harmonics) shape invariant under Fourier transformation, while the spherical Hankel transform (113) of the radial function ϒ_ν_(κ_β_ξ) remains.
